# Review on Techniques for Thermal Characterization of Graphene and Related 2D Materials

**DOI:** 10.3390/nano11112787

**Published:** 2021-10-21

**Authors:** Jing Liu, Pei Li, Hongsheng Zheng

**Affiliations:** College of New Materials and New Energies, Shenzhen Technology University, Shenzhen 518116, China; 2070413005@stumail.sztu.edu.cn (P.L.); 20183280058@stumail.sztu.edu.cn (H.Z.)

**Keywords:** optothermal Raman technique, thermal transport, 2D materials

## Abstract

The discovery of graphene and its analog, such as MoS_2,_ has boosted research. The thermal transport in 2D materials gains much of the interest, especially when graphene has high thermal conductivity. However, the thermal properties of 2D materials obtained from experiments have large discrepancies. For example, the thermal conductivity of single layer suspended graphene obtained by experiments spans over a large range: 1100–5000 W/m·K. Apart from the different graphene quality in experiments, the thermal characterization methods play an important role in the observed large deviation of experimental data. Here we provide a critical review of the widely used thermal characterization techniques: the optothermal Raman technique and the micro-bridge method. The critical issues in the two methods are carefully revised and discussed in great depth. Furthermore, improvements in Raman-based techniques to investigate the energy transport in 2D materials are discussed.

## 1. Introduction

Since the discovery of graphene and other 2D materials such as MoS_2_, various properties of 2D materials have been intensively studied [1,2,3,4,5]. The ultra-high thermal conductivity of graphene has led to extensive experimental research and theoretical simulations about the energy transport in it in past decades [3,6,7]. The thermal transport in other 2D materials also gains much interest for its promising applications [8,9]. However, compared with the thermal conductivity (*κ*) obtained from simulations, *κ* obtained from experiments shows large discrepancies. For example, *κ* of suspended single layer graphene (SLG) ranges from 1100 to 5300 W/m·K [3,10], depending on the thermal characterization method and the fabrication method of graphene. *κ* of supported graphene drops to hundreds W/m·K, which is also related to the substrate [6,11,12]. It is well accepted that *κ* of supported graphene is suppressed due to phonon leakage [6,13]. Table 1 summarizes *κ* of suspended and supported graphene by different experimental methods. A large discrepancy among *κ* can be observed. This discrepancy arises from the characterization methods and the quality of the graphene. The thermal characterization techniques of 2D materials include the optothermal Raman technique [3,11,14], micro-bridge method [6,9], time-domain thermoreflectance (TDTR) [15] and Johnson noise thermometry [16]. In this paper, we will focus on the optothermal Raman technique and the micro-bridge method. The critical issues faced in the above two methods will be discussed in depth. The issues in the optothermal Raman technique include the accuracy of the laser power absorbed by 2D materials, stress effect and inter-phonon branch nonequilibrium. When it comes to the micro-bridge method, the thermal resistance of the 2D materials should be properly chosen to guarantee measurement accuracy. These issues undermine the measurement accuracy in thermal transport characterization of 2D materials [17].

## 2. Raman Optothermal Method

**Figure 1:**
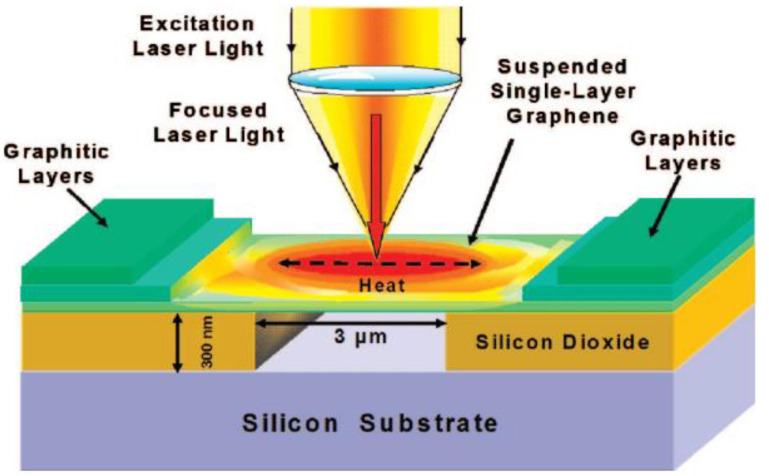
Schematic of the optothermal Raman technique. Reprinted with permission from ref. [3]. Copyright 2008 American Chemical Society.

The characteristic peaks in Raman spectra of the 2D materials have strong temperature dependence. It is possible to make use of the Raman spectra to characterize the thermal transport in 2D materials [8,21,22,23,24]. Balandin first developed the confocal Raman spectroscopy to measure *κ* of suspended graphene [3]. Schematic of the optothermal Raman technique is shown in Figure 1. As the laser spot is much smaller than the suspended graphene size, the heat is propagating radially to the edges. By obtaining the Raman shift temperature coefficient (*χ_T_*) and the Raman shift power coefficient (*χ_P_*) of *G* peak, the thermal conductivity of graphene can be expressed as *κ* = *χ_T_*(*L*/2*hW*)/*χ_P_* [3]. Here, *L*, *h* and *W* are the distance from the hot spot to the heat sink, thickness of SLG and width of the sample, respectively. The optothermal Raman technique is proven to be powerful and widely used in characterizing the energy transport in 2D materials [25]. Advantages of the optothermal Raman method include minimal sample preparation, high spatial resolution and material specificity [17]. However, it is important to point out that several critical issues should be carefully considered regarding the utilization of the Raman optothermal method.

One important parameter in the deduction of *κ* of graphene is the laser power (*P*) absorbed by the graphene. Usually, two methods are employed to obtain *P*. One is calculating the absorbed power based on the optical properties [3]. It is well accepted that the absorption coefficient (*α_G_*) of SLG is 2.3% [2]. Thus, *P* can be described as *P* = *I*_0_*A*(1 − exp(−*α_G_δ*)), where *I*_0_ is the laser intensity on the surface, *A* is the illuminated area and *δ* is the thickness of SLG [3]. However, the *α_G_* is easily affected by many factors, such as the wrinkles and the strain [26]. The optical properties can vary greatly from sample to sample, resulting in uncertainty in the laser absorption. In supported graphene, the laser absorption is significantly affected by the interface-induced optical interference [27], which leads to great uncertainty in the laser absorption calculation. Another method is directly measuring the transmitted power. Thus, the absorbed power can be obtained by subtracting the transmitted power from the total incident laser power. However, a very small proportion of the incident laser power is absorbed by graphene. Thus, even very little variation in the transmitted power can lead to great uncertainty in the absorbed power. The uncertainty in *P* will affect the accuracy of *χ_P_*, which further introduces uncertainty into the derivation of *κ*. It is difficult to determine the uncertainty of *P.* If there is 10% uncertainty in *χ_P_*, 10% uncertainty will be introduced to *κ.*

Another source of uncertainty in the optothermal Raman method is the stress effect in the graphene. During the temperature coefficient calibration, the whole sample is in thermal equilibrium. However, the graphene experiences thermal nonequilibrium in the experiment. This leads to the different stress effects in the graphene during the calibration process and experiment. Thus, the temperature probed by Raman spectroscopy is not precise, which further introduces uncertainty into *κ* determination [28]. Apart from this, the Raman spectroscopy actually detects the temperature of the optical phonons, which is easily affected by the thermomechanical stress. The thermomechanical stress in few layers graphene (FLG) alters the interatomic-potential, which affects the energy of the optical phonons [17]. Theoretical simulation shows that the uncertainty caused by the thermomechanical stress in *κ* of FLG can be higher than 20% [17].

It is critically important to point out that the Raman optothermal method is based on an assumption that different phonon branches are in thermal equilibrium under the photon excitation. However, Ruan et al., first reported that the phonon branches were in strong thermal nonequilibrium by employing the density functional perturbation theory (DFPT) [29]. For example, the steady-state temperature of transverse optical phonons (*T_TO_*) can be 14.8% higher than that (*T_ZA_*) of out-of-plane acoustic (ZA) phonons at the center of the SLG [30]. By using the multitemperature model (MTM) developed by Ruan et al., the predicted *κ* of SLG is increased by 67% [30]. Ruan’s theoretical simulation enlightened the experimental work about the thermal nonequilibrium among phonon branches. The temperature differences between different phonon branches in 2D materials under Raman excitation were first verified and detected by Wang’s group by experiment [31]. Wang et al., distinguished the temperatures of optical (*OP*) and acoustic (*AP*) phonons under phonon excitation in 2D materials by constructing steady and nanosecond (ns) inter-phonon branch energy transport states [31]. By developing the nanosecond energy transport state-resolved Raman (ns ET-Raman) technique, the temperature difference (Δ*T_OP-AP_*) between *OP* and *AP* is reported to be 30% larger than the Raman-probed temperature rise in MoS_2_ [31].

**Figure 2:**
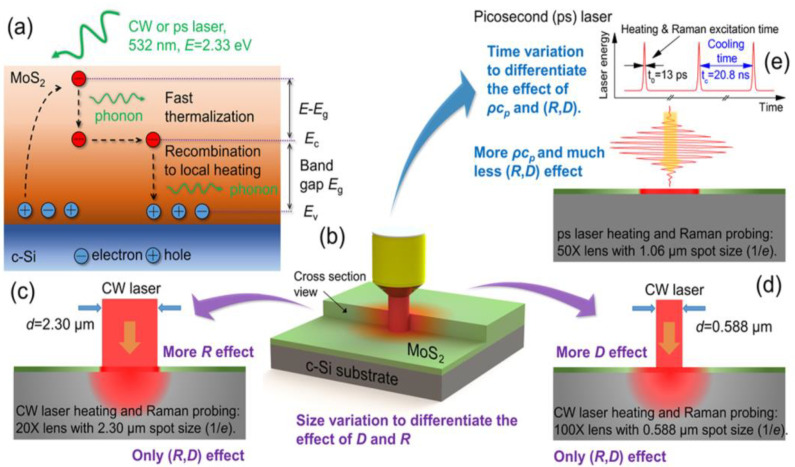
(**a**) Hot carrier diffusion in MoS_2_/c-Si under laser illumination (not to scale): *E_v_* and *E_c_* are the valence band and conduction band, respectively, *E_g_* is the bandgap of MoS_2_, *E* is the photon energy of the incident laser; (**b**) schematic of the experiment setup (not to scale): *ρc_p_* is the volumetric heat capacity of the sample; (**c**,**d**) continuous wave (CW) laser is used to heat the sample under 20× and 100× objective lens to achieve different hot aera size: *R* and *D* are the interface thermal resistance and hot carrier diffusion coefficient, respectively; (**e**) picosecond pulsed laser is used under 50× objective lens to achieve zero thermal transport state. Reprinted with permission from ref. [27]. Copyright 2017 American Chemical Society.

In most research about the energy transport in 2D materials by Raman spectroscopy, the hot carrier diffusion effect is not considered, which is more prominent as the laser spot size is smaller than 0.5 μm [28]. Here, we look at a MoS_2_/c-Si structure and discuss what will happen after the laser illumination on the sample. Figure 2a shows the physical principle of the electrons and holes diffusion under the laser illumination. Subsequent to laser irradiation, the electrons and holes are generated by absorbing photons. In extremely short time (~ps), the excess energy (Δ*E* = *E* – *E_g_*) of the electrons will quickly dissipate to other unexcited electrons and the lattice. Then, the electrons and holes (hot carriers) diffuse and recombine, releasing the energy by scattering with the optical phonons. This leads to a much larger thermal source area than the excitation spot. The specific process is described in detail in the reference [32]. The electron-hole diffusion has a negligible effect on the thermal conduction in suspended 2D materials [33]. However, the effect of the hot carrier diffusion should be carefully handled when determining the interface thermal energy transport between graphene and substrate.

## 3. Micro-Bridge Method

**Figure 3:**
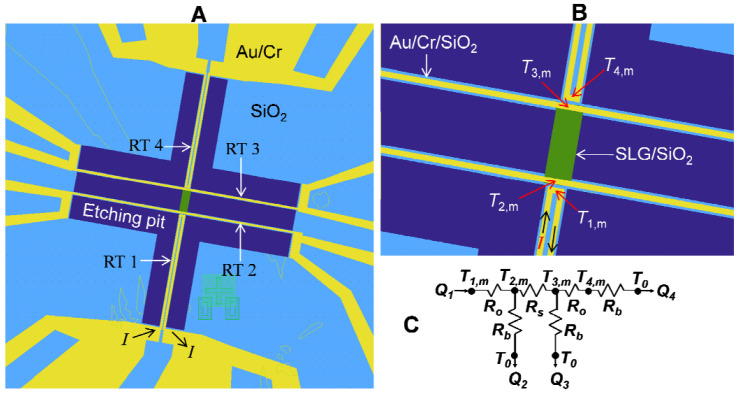
(**A**,**B**) SEM images of micro-bridge method setup; (**C**) circuit of the thermal resistance. Reprinted with permission from ref. [6]. Copyright 2010 American Association for the Advancement of Science.

Shi Li et al., first employed the micro-bridge method to measure *κ* of graphene supported on amorphous SiO_2_ [6]. The schematic of the experiment is shown in Figure 3. There are four Au/Cr resistance thermometer (RT) lines in the setup. The two straight RT lines (RT2 and RT3) cover the two ends of the graphene. The U-shaped RT1 and RT4 separate from both the graphene and the RT2 and RT3. During the experiment, the RT1 is self-heated by applying current into it. Based on the thermal resistance circuit shown in Figure 3c, thermal resistance (*R_s_*) of the central beam including both graphene and SiO_2_ can be expressed as: $R_{s}=R_{b}\frac{\Delta T_{2,m}-\Delta T_{3,m}}{\Delta T_{3,m}+\Delta T_{4,m}}$ [6]. Here, Δ*T_j,m_* (*j* = 2,3,4) is the temperature rise at the middle point of RT. *R_b_* is the thermal resistance of each RT line with the SiO_2_ beam. By measuring the *Rs* before and after removing the graphene, *κ* of graphene is determined. The accuracy of the micro-bridge method is guaranteed only as *R_s_* is comparable to *R_b_*. As *κ* of the graphene is low or the length of the graphene/SiO_2_ beam is longer than tens of μm, *R_s_* will be much larger than *R_b_*. Thus, the heat conducting into the graphene/SiO_2_ beam will be very small, which will lead to great uncertainty in the measurement of Δ*T_m_*. This will further increase the uncertainty in the determination of *κ* of graphene. Overall, the micro-bridge method is feasible in principle, but its measurement accuracy is guaranteed when the sample has proper thermal resistance. In addition, it is technically challenging and time consuming to fabricate the whole measurement device.

**Figure 4:**
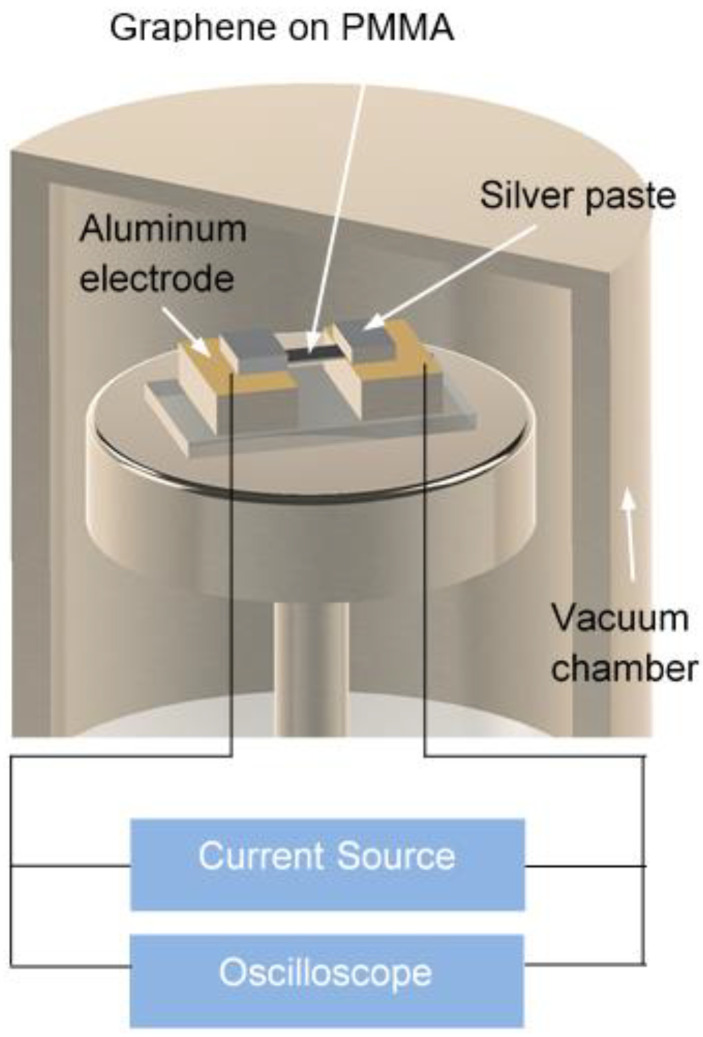
Experiment setup of the TET technique. Reprinted from ref. [12].

The sample size tested by the Raman spectroscopy method and micro-bridge method is at the level of several μm. However, the mean free path (MFP) of phonons in graphene can be as long as hundreds of micrometers [34], surpassing the sample size. This will result in a strong phonon-edge scattering effect in the graphene. To investigate the intrinsic *κ* of graphene without or with minimal phonon-edge scattering, Liu et al., first developed the differential transient electrothermal technique (TET) to characterize *κ* of giant graphene supported by polymethyl methacrylate (PMMA) [12]. The experiment setup is shown in Figure 4. The graphene supported by PMMA is suspended between two electrodes. During the experiment, the whole sample is fed through with step current to induce Joule heating in it. The voltage evolution (*V*(*t*)) of the sample is recorded by an oscilloscope. The thermal diffusivity (*β*) of the whole sample can be obtained by fitting the *V*(*t*)*~t* curve. *κ_eff_* of the whole sample is calculated as *κ_eff_* = *β·ρc_p_*. Through simulation, it is found that the interface thermal resistance between graphene and PMMA is negligible in *κ_eff_*. Thus, *κ_eff_* can be written as *κ_eff_* = *f*(*κ_p_*, *κ_g_*, *δ_p_*, *δ_g_*). Here, the subscripts *p* and *g* indicate PMMA and graphene, respectively. *δ* is thickness. *κ* of SLG supported by PMMA was determined to be 365 W/m·K [12], which is only 60% of *κ* of graphene supported by amorphous SiO_2_ [6]. The authors attributed the low thermal conductivity of SLG on PMMA to abundant carbon atoms in the PMMA [12]. The abundant carbon atoms lead to a strong phonon scattering effect between SLG and PMMA.

## 4. Improvements in the Optothermal Raman Technique

In order to resolve the challenges mentioned above in the optothermal Raman technique, various Raman-based techniques, including frequency-domain energy transport state-resolved Raman (FET-Raman) [35,36] and energy transport state-resolved Raman (ET-Raman) [33], were developed. They can free the Raman thermometry from the laser absorption and the temperature coefficient calibration. In the FET-Raman experiment, the 2D material experiences two energy transport states. The first one is the steady-state heating induced by a CW laser. By varying the incident laser power (*P*), the Raman shift power coefficient (RSC) *χ_steady-state_* = ∂*ω*/∂*P* = *α*·(∂*ω*/∂*T*)·*f*_1_(*κ*) is obtained. Here, *α* is the laser absorption coefficient, ∂*ω*/∂*T* is the Raman shift temperature coefficient and *κ* is the in-plane thermal conductivity. The second energy transport state is the transient-state heating induced by a square wave-modulated CW laser. Similarly, an RSC can be obtained: *χ_transient_* = ∂*ω*/∂*P* = *α*·(∂*ω*/∂*T*)·*f*_2_(*κ*, *ρc_p_*), where *ρc_p_* is the volumetric heat capacity of the sample. Since the thermal diffusion lengths in two energy states are different, a normalized RSC parameter can be defined as Θ = *χ_transient_*/*χ_steady-state_* = *f*_3_(*κ*, *ρc_p_*) [35]. Thus, the effect of *α* and ∂*ω*/∂*T* is eliminated. By interpolating the Θ obtained from the experiment into the Θ–*κ* curve obtained from a 3D numerical simulation of the MoSe_2_ sample, *κ* of MoSe_2_ is determined. The feasibility of the FET-Raman technique has been verified by employing the FET-Raman to determine *κ* of MoSe_2_ and the anisotropic thermal conductivities of carbon fibers [35,36].

Inspired by the theoretical simulation by Ruan et al. [29,30,37], Wang et al., first developed ns ET-Raman to explore the inter-phonon branch non-equilibrium effect in 2D materials under photon excitation [31]. Under CW laser excitation, the local temperature rise (Δ*T_m_*) consists of the temperature rise of acoustic phonons (Δ*T_AP_*) and the temperature difference between optical phonons and acoustic phonons (Δ*T_OA_*). Δ*T_m_* can be expressed as:

(1) $$\Delta T_{m}=\Delta T_{AP}+\Delta T_{OA}=\Delta T_{AP}+\delta I/G_{pp}$$

Here, *I* is the intensity of the absorbed laser at location *r*. *δ* (0 < *δ* < 1) is the portion of the laser energy transfer from optical phonons (*OP*) to acoustic phonons (*AP*). *G_pp_* is the coupling factor between *OP* and *AP*. Furthermore, the Raman intensity weighted temperature rise ($\Delta {{\bar{T}}_{m}|}_{CW}$) probed by the Raman spectroscopy can be expressed as [21]:

(2) $$\Delta {\bar{T}}_{m}{|}_{CW}=\frac{\iint \Delta T_{m}I_{CW}e^{-z/\tau_{L}}2\pi rdrdz}{\iint {}_{CW}e^{-z/\tau_{L}}2\pi rdrdz}\;=\Delta {\bar{T}}_{OA}{|}_{CW}+\Delta {\bar{T}}_{AP}{|}_{CW}=\frac{1}{3}\times \frac{I_{0}}{\tau_{L}}\times \frac{\delta }{G_{pp}{|}_{CW}}+\Delta {\bar{T}}_{AP}{|}_{CW}$$

Here, *I_CW_* is the laser intensity distribution of the CW laser. $\Delta {{\bar{T}}_{OA}|}_{CW}$ and $\Delta {{\bar{T}}_{AP}|}_{CW}$ are the Raman intensity weighted temperature difference between *OP* and *AP* and the temperature rise of the acoustic phonons, respectively. *I*_0_ is the absorbed laser power per unit area, and *τ_L_* is the absorption depth. Before determining the percentages of $\Delta {{\bar{T}}_{OA}|}_{CW}$ and $\Delta {{\bar{T}}_{AP}|}_{CW}$ in $\Delta {{\bar{T}}_{m}|}_{CW}$, $G_{pp}{|}_{CW}$ should be figured out first by experiment. By constructing a 3D heat conduction model for a 55nm-thick MoS_2_, $\Delta {{\bar{T}}_{AP}|}_{CW}$ is obtained as $\Delta {{\bar{T}}_{AP}|}_{CW}=0.94+2.86e^{-1.65r_{0}}$. *r*_0_ is the radius of the laser spot. The Raman shift is proportional to the temperature rise caused by unit power; thus, the Raman shift coefficient *χ_CW_* can be expressed as [31]:

(3) $$\chi_{CW}=A\times [(0.94+2.86e^{-1.65r_{0}}+\frac{1}{3}\times \frac{P}{\pi r_{0}^{2}\tau_{L}}\times \frac{\delta }{{G_{pp}|}_{CW}})]/P$$

Here, *A* is a constant. In the experiment, three objective lenses (20×, 50× and 100×) are employed to obtain the $\chi_{CW}{-r}_{0}$ relationship. By fitting $\chi_{CW}{-r}_{0}$ relationship with Equation (3), $G_{pp}{|}_{CW}$ can be determined. Furthermore, the Raman intensity weighted temperature rise $\Delta {{\bar{T}}_{OA}|}_{CW}$ and $\Delta {{\bar{T}}_{AP}|}_{CW}$ can be figured out through the 3D conduction numerical calculation. It is found that the temperature difference between *OP* and *AP* accounts for more than 30% of the temperature rise detected by Raman [31]. Zobeiri et al., further characterized the thermal nonequilibrium between *OP* and *AP* under photon excitation in graphene paper [38]. The Raman intensity weighted temperature rise of *OP* is found to be 82.1% higher than that of *AP* under 100× laser heating [38], which indicates the importance of taking interphonon thermal nonequilibrium effects into consideration in the optothermal Raman technique.

To consider the hot carrier diffusion effect in the thermal transport in 2D materials, Yuan et al., first developed the ET-Raman to determine the interface thermal resistance and hot carrier diffusion coefficient in MoS_2_ supported by c-Si [27,33]. The ET-Raman includes two energy transport states: the zero thermal transport state and the steady-state thermal transport. The zero thermal transport state is obtained by applying a picosecond pulsed laser under a 50× objective lens. In an extremely short pulse (~13 ps) time, only the fast thermalization process happens, so the heat conduction in the lattice can be neglected. By varying the laser power, the RSC *χ_ps_* = ∂*ω*/∂*P* is obtained, which is more affected by the *ρc_p_* rather than by the hot carrier diffusion coefficient (*D*) and interface thermal resistance (*R*). In the steady-state thermal transport, the RSC under 20× and 100× objective lenses are obtained by applying the *CW* laser. Both *χ_CW_*_20_ under the 20× objective lens and *χ_CW_*_100_ under the 100× objective lens carries the information of *D* and *R*. However, *χ_CW_*_20_ is more affected by *R*, while *χ_CW_*_100_ is more affected by *D*. Here, the normalized RSC is defined as Θ_1_ = *χ_CW_*_20_/*χ_ps_* and Θ_2_ = *χ_CW_*_100_/*χ_ps_*. By simulating a 3D heat conduction model in the sample, the RSC contour with *R* and *D* as variables is obtained. The cross point of the Θ_1_ curve and Θ_2_ curve gives the value of *R* and *D*.

## 5. Conclusions

In summary, though facing several critical problems, the optothermal Raman technique and micro-bridge method still show suitability and feasibility in energy transport characterization in 2D materials. Much pioneering work about the thermal non-equilibrium in different phonon branches has been reported. However, the physical model and data fitting used in the pioneering work still suffer great uncertainties and make the study rather semi-quantitative. Secondly, past work has studied the thermal nonequilibrium in suspended 2D materials. However, for supported 2D materials, how the interface resistance between 2D material and substrate affects the *OP*-*AP* thermal nonequilibrium is unclear. Further work can be focused on the *OP*-*AP* thermal nonequilibrium in supported 2D materials.

## Figures and Tables

**Table 1:** Thermal conductivity of graphene obtained by experiments.

*κ* (W/m·K)	Method	Brief Description	References
~3000–5000	Raman optothermal	Suspended SLG ^1^, exfoliated	[3]
2500 + 1100/ − 1050	Raman optothermal	Suspended SLG, CVD ^2^	[11]
400–1800	Raman optothermal	Suspended SLG with crystal lattice defects	[18]
730–880 ± 60	Micro-bridge	Suspended bilayer graphene, PMMA ^3^ residues on the surface	[19]
1896 ± 390	Raman optothermal	Suspended bilayer graphene	[20]
365	Transient Thermoelectrical technique (TET)	Supported SLG on PMMA, giant scale, CVD	[12]
370 + 650/ − 320	Raman optothermal	Supported SLG on copper, CVD	[11]
600	Micro-bridge	Supported SLG on amorphous SiO_2_^4^, exfoliated	[6]

^1^ SLG: Single layer graphene. ^2^ CVD: Chemical vapor deposition. ^3^ PMMA: Polymethyl methacrylate. ^4^ SiO_2_: Silicon dioxide.

## Data Availability

No new data were created or analyzed in this review. Data sharing is not applicable to this article.
